# Effects of DNA Methylation and Chromatin State on Rates of Molecular Evolution in Insects

**DOI:** 10.1534/g3.115.023499

**Published:** 2015-12-02

**Authors:** Karl M. Glastad, Michael A. D. Goodisman, Soojin V. Yi, Brendan G. Hunt

**Affiliations:** * School of Biology, Georgia Institute of Technology, Atlanta, Georgia 30332; † Department of Entomology, University of Georgia, Griffin, Georgia 30223

**Keywords:** coding sequence evolution, cytosine methylation, histone modifications, *Drosophila melanogaster*, *Camponotus floridanus*

## Abstract

Epigenetic information is widely appreciated for its role in gene regulation in eukaryotic organisms. However, epigenetic information can also influence genome evolution. Here, we investigate the effects of epigenetic information on gene sequence evolution in two disparate insects: the fly *Drosophila melanogaster*, which lacks substantial DNA methylation, and the ant *Camponotus floridanus*, which possesses a functional DNA methylation system. We found that DNA methylation was positively correlated with the synonymous substitution rate in *C. floridanus*, suggesting a key effect of DNA methylation on patterns of gene evolution. However, our data suggest the link between DNA methylation and elevated rates of synonymous substitution was explained, in large part, by the targeting of DNA methylation to genes with signatures of transcriptionally active chromatin, rather than the mutational effect of DNA methylation itself. This phenomenon may be explained by an elevated mutation rate for genes residing in transcriptionally active chromatin, or by increased structural constraints on genes in inactive chromatin. This result highlights the importance of chromatin structure as the primary epigenetic driver of genome evolution in insects. Overall, our study demonstrates how different epigenetic systems contribute to variation in the rates of coding sequence evolution.

The evolutionary rates of protein-coding genes span multiple orders of magnitude within the genome of a single taxon (Wolf *et al.* 2009). Determining the functional, structural, and regulatory sources of variation in constraints on protein-coding sequences has been central to advancing our understanding of evolution at the molecular level (Koonin and Wolf 2010). Accordingly, a large and growing body of research has revealed fundamental insights into near-universal constraints on protein-coding sequence evolution (Pal *et al.* 2006; Koonin and Wolf 2010). These constraints include the essentiality of a protein to organismal survival (Wall *et al.* 2005; Liao *et al.* 2006), gene expression level (Drummond and Wilke 2008), gene expression pattern (Duret and Mouchiroud 2000; Hunt *et al.* 2013c), and gene compactness (Eisenberg and Levanon 2003; Carmel *et al.* 2007).

In addition, chromatin structure has recently been investigated as a factor influencing molecular evolution. Associations between chromatin structure and constraints on gene evolution can arise as a byproduct of the link between chromatin structure and gene expression patterns (Prendergast *et al.* 2007; Filion *et al.* 2010; Kharchenko *et al.* 2011). Variation in mutation rate and sequence constraints are also linked to nucleosome positioning and chromatin accessibility (Prendergast *et al.* 2007; Prendergast and Semple 2011; Tolstorukov *et al.* 2011; Schuster-Bockler and Lehner 2012; Langley *et al.* 2014; Makova and Hardison 2015). However, the relationship between chromatin structure and molecular evolution has yet to be investigated in insect genomes, where evolutionary variation in DNA methylation provides the opportunity to disentangle the relative effects of DNA methylation and other epigenetic marks (Glastad *et al*. 2011). Specifically, although functional intragenic DNA methylation exists in multiple insect species, other insect groups, such as flies, exhibit evidence of a near-complete loss of DNA methylation (Zemach *et al.* 2010; *cf*. Takayama *et al.* 2014).

Our primary interest in undertaking this study was to better understand how DNA methylation and chromatin structure affect genome evolution. Studies in plants and animals have shown that variation in intragenic DNA methylation affects gene regulation by altering local chromatin and the rate of elongation of RNA pol II (Zilberman *et al.* 2007; Maunakea *et al.* 2013). Similarly, the regulatory roles of histone modifications are known to include the mediation of binding affinities of protein complexes, such as those related to transcriptional and splicing machinery, as well as the direct alteration of local chromatin structure (Luco *et al.* 2010; Bell *et al.* 2011; Bintu *et al.* 2012). Together, DNA methylation and histone modifications interact to contribute to a multifaceted epigenetic landscape in eukaryotic cells (Cedar and Bergman 2009). For example, in insects with functional DNA methylation systems, the targeting of DNA methylation has been shown to exhibit striking associations with multiple histone modifications that are, in turn, linked to active transcription (Nanty *et al.* 2011; Hunt *et al.* 2013b; Glastad *et al.* 2015).

Although DNA methylation is primarily targeted to cytosines at cytosine-phosphate-guanine (CpG) dinucleotides in eukaryotes (Klose and Bird 2006), the localization of DNA methylation varies substantially among taxa. In vertebrate animals, DNA methylation is present globally within the genome, with only small regions of unmethylated DNA found largely in gene promoters (Suzuki and Bird 2008). In contrast, the genomes of invertebrates exhibit relatively sparse levels of DNA methylation, present almost exclusively in genes (Suzuki and Bird 2008; Feng *et al.* 2010; Zemach *et al.* 2010). DNA methylation is known to increase the mutation rate of affected cytosines, particularly in intronic and intergenic regions (Bird 1980; Elango *et al.* 2008; Mugal and Ellegren 2011; Drewell *et al.* 2014). Despite this mutational effect, the presence of DNA methylation in gene bodies is paradoxically associated with protein conservation (Takuno and Gaut 2012; Chuang and Chiang 2014; Glastad *et al.* 2014), likely due to several confounding attributes of methylated genes, such as elevated selective constraint and decreased nucleosome occupancy (Takuno and Gaut 2012).

Here, we investigate the relationships between epigenetic marks and coding sequence evolution in two insects, the fruit fly *Drosophila melanogaster*, and the carpenter ant *Camponotus floridanus*. Distinct chromatin states have been well characterized in *D. melanogaster* (Filion *et al.* 2010; Kharchenko *et al.* 2011) and, more recently, genome-wide spatial profiles of many histone modifications have been examined in the ant *C. floridanus* (Simola *et al.* 2013b). Importantly, a comparison of these taxa provides a novel opportunity to determine the contribution of DNA methylation to coding sequence evolution because *C. floridanus* exhibits substantial genomic DNA methylation (Bonasio *et al.* 2012) but *D. melanogaster* does not (Zemach *et al.* 2010; *cf*. Takayama *et al.* 2014). Therefore, our investigation allows us to isolate the effects of DNA methylation on gene evolution and provide direct insight into how epigenetic information affects molecular evolution.

## Materials and Methods

### Molecular evolution

Single-copy orthology was assigned (i) across seven ant species (*C. floridanus*, *Harpegnathos saltator*, *Linepithema humile*, *Pogonomyrmex barbatus*, *Solenopsis invicta*, *Acromyrmex echinator*, and *Atta cephalotes*), and (ii) between *C. floridanus* and *D. melanogaster* by orthoDB (Waterhouse *et al.* 2011; Simola *et al.* 2013a).

Multiple sequence alignment was performed with PRANK (Löytynoja and Goldman 2005), as implemented by GUIDANCE (Penn *et al.* 2010). PhyML (Guindon *et al.* 2010) was used to impute trees from multiple sequence alignments, modifying branch lengths and rate variables, but keeping topology the same as input trees. Gblocks (Talavera and Castresana 2007) was then used to filter alignment columns, using default settings, prior to further analyses.

Coding sequence substitution rates for *D. melanogaster*, as summed over species from the *D. melanogaster* species subgroup, were calculated previously (Drosophila 12 Genomes Consortium 2007). Substitution rates for ants were averaged across all aligned codons for a given protein, with free dN/dS ratios for each branch, using PAML with the F3×4 codon model (Yang 2007). We filtered out genes for which dN or dS values were greater than 14 across the seven ant tree, as well as genes that had an aligned length of less than 50 codons. In order to mask CpG dinucleotides for an additional analysis, a separate dataset was produced wherein alignment columns with a CpG in any of the aligned species were masked before running PAML.

### Chromatin immunoprecipitation sequencing (ChIP-seq)

We used ChIP-seq data that were generated previously for *C. floridanus* (Simola *et al.* 2013b). We remapped these data to the *C. floridanus* genome (Cflo_3.3) using bowtie (Langmead *et al.* 2009), after filtering for adapter contamination and read quality using Trimmomatic (Bolger *et al.* 2014). We allowed one mismatch in the “seed” region, and only accepted the most valid alignment for each mapping read.

MACS2 (Zhang *et al.* 2008) was then used to estimate the read enrichment relative to an input control (as well as bulk histone H3 profiles for histone modifications to histone H3) for each ChIP library after removal of duplicate reads. We only allowed one of each duplicated read when running MACS in an effort to minimize bias introduced through PCR amplification. ChIP enrichment scores were assigned to a coding sequence (CDS) as fold enrichment value over normalized read counts overlapping the given CDS for merged libraries from major workers, minor workers, and males (Simola *et al.* 2013b).

ChIP-seq data from *D. melanogaster* embryos were obtained for each histone modification from modEncode (Celniker *et al.* 2009; modENCODE ID numbers: 3955, 4120, 4938, 4939, 4950, 5092, 5096, 5103), and mapped to *D. melanogaster* genome build r5.42 CDS annotations. *D. melanogaster* ChIP enrichment scores were assigned to a CDS following the methods described for *C. floridanus*.

### Whole genome bisulfite sequencing (WGBS)

We calculated fractional DNA methylation levels, as averaged across all CpG dinucleotides from a given coding sequence, following methods described in detail previously (Hunt *et al.* 2013b). We used previously generated WGBS data from *C. floridanus* (Bonasio *et al.* 2012), accessed from the NCBI GEO database (GSE31577). DNA methylation levels were assessed for merged libraries from queens, workers, and males.

### Transcriptome sequencing (RNA-seq)

RNA-seq reads from adult *C. floridanus* were generated previously (Bonasio *et al.* 2012). We filtered (Bolger *et al.* 2014) and aligned these reads to the *C. floridanus* genome (v3.3) using tophat (Trapnell *et al.* 2009). Cufflinks (Roberts *et al.* 2011) was then run with multiread-correction, fragment bias correction, and upper quartile normalization. Cuffdiff (Roberts *et al.* 2011) fpkm values from queen, worker, and male libraries were averaged to represent *C. floridanus* gene expression level.

We used *D. melanogaster* RNA-seq ‘modENCODE Transcriptome v2 Expression Scores’, obtained from the Berkeley Drosophila Genome Project (http://fruitfly.org/sequence/download.html; Celniker *et al.* 2009). The mean of gene expression levels from 4-day posteclosion mated male and female heads was used to represent *D. melanogaster* gene expression level.

### Gene structure and annotation

Mean intron and exon sizes were calculated using *C. floridanus* 3.3 gene models and *D. melanogaster* flybase v5.42 (FB2011_10) gene models.

*C. floridanus* gene ontology (GO) annotations were assigned using Blast2GO (Conesa *et al.* 2005). Blast2GO’s inbuilt ‘gossip’ package was used to test for enrichment using a Fisher’s exact test, correcting for multiple testing using a Benjamini-Hochberg false discovery rate (FDR). Significantly enriched terms (FDR *P* < 0.05) were reduced to the most specific enriched terms for presentation.

### Statistical analyses

Prior to linear model analysis, all data were log-transformed (following the addition of 0.0001 to prevent discarding zero values, except in the case of intron length, for which a value of 1 was added prior to log-transformation to reduce the degree to which genes lacking introns were outliers), and then standardized (mean = 0, standard deviation = 1) in the R statistical computing environment (R Development Core Team 2013). Multiple linear regression models, which incorporated the weighted effects of all included variables, were fitted with the ‘lm’ function in R, and confidence intervals for model parameters were obtained with the ‘confint’ function in R.

The JMP statistical software package (SAS Institute Inc, Cary, NC) was used to perform principal component analysis, which directly addresses the issue of collinearity among variables, and to calculate Pearson’s correlations. We found that multiple linear regression models using substitution rates summed over seven ant species explained greater variance in dependent variables than those measured for the *C. floridanus* branch alone (seven ant dS *R*^2^ = 0.28, *C. floridanus* branch dS *R*^2^ = 0.11; seven ant dN *R*^2^ = 0.19, *C. floridanus* branch dN *R*^2^ = 0.17). Thus, we chose to use dN and dS values summed over the seven ant tree.

### Data availability

Data used to perform statistical analyses are available in Supporting Information, File S2. Raw sequencing reads are publicly available, as described in the respective source publications, cited above.

## Results and Discussion

### Coding sequence evolution in the presence and absence of DNA methylation

**Figure 1:**
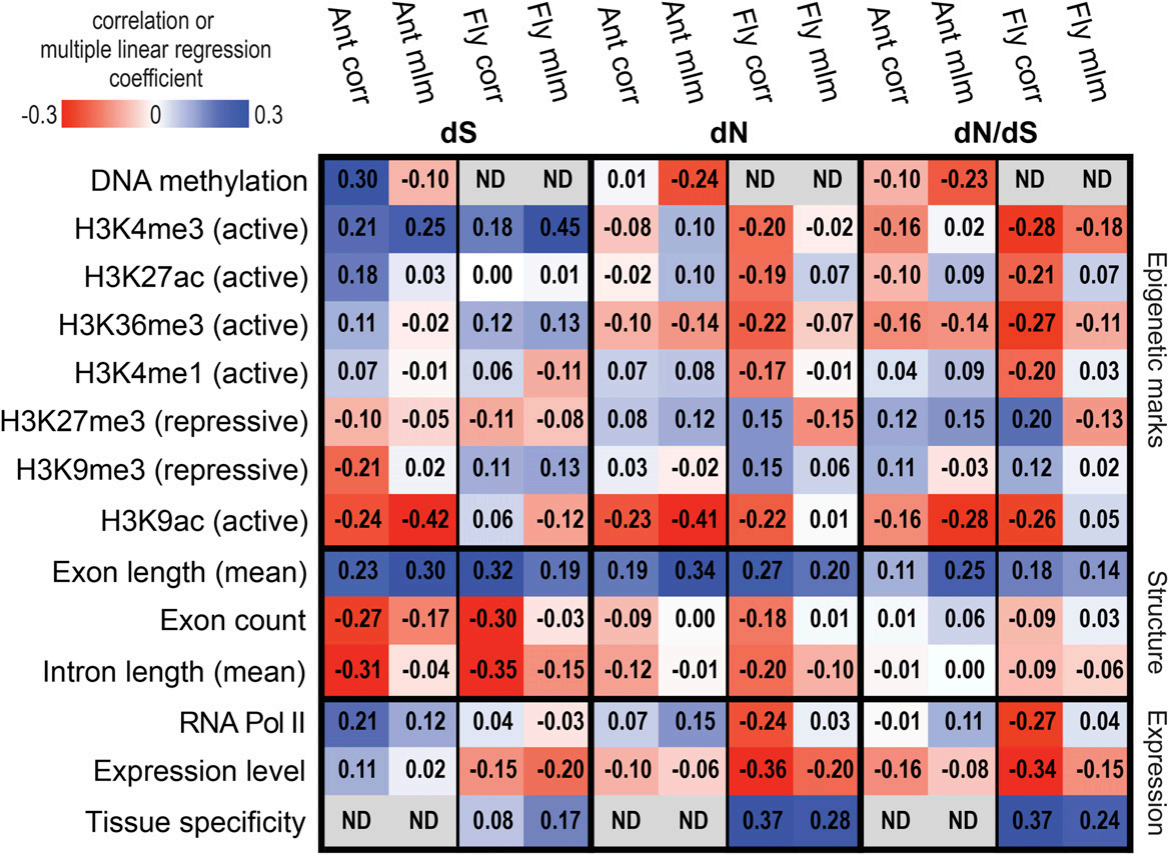
Correlation coefficients (corr) and multiple linear regression model coefficients (mlm) between sequence substitution rates and gene characteristics in the ant *Camponotus floridanus*, and the fly *Drosophila melanogaster*. ‘Active’ and ‘repressive’ histone modification designations indicate associations with active transcription and repression of transcription in *D. melanogaster* (Kharchenko *et al.* 2011). *C. floridanus* n = 4984 genes, *D. melanogaster* n = 7396 genes. ac, acetylation; H3, histone H3; K, lysine; me1, monomethylation; me3, trimethylation; Pol, Polymerase.

Our first goal in this study was to understand how DNA methylation affects rates of molecular evolution. In *C. floridanus*, we observed that DNA methylation was the second largest negative correlate of both dN and dN/dS when controlling for other factors using multiple linear regression (Figure 1, Figure S2, Table S3, and File S1). This association is consistent with the preferential targeting of DNA methylation to constitutively expressed, phylogenetically conserved genes in insect genomes (Sarda *et al.* 2012; Hunt *et al.* 2013b; Glastad *et al.* 2014).

We also observed a negative association between dS and DNA methylation in *C. floridanus*, when considered in a multiple linear regression framework (Figure 1 and Figure S2). This finding was surprising given evidence that DNA methylation results in elevated mutation rates in mammals (Elango *et al.* 2008; Mugal and Ellegren 2011), as well as in many insects (Glastad *et al.* 2011, 2013; Drewell *et al.* 2014), which exhibit much lower levels of DNA methylation than vertebrates (Zemach *et al.* 2010). However, we did observe a striking and positive association between DNA methylation and dS according to a pairwise correlation analysis (Figure 1 and Figure S1).

One possible explanation for the discrepancy between our multiple linear regression analysis and pairwise correlation analysis is that one or more included variables exhibits collinearity with DNA methylation, thus masking the relationship between dS and DNA methylation. Such collinearity is not surprising, given that multiple histone modifications are tightly associated with DNA methylation in insect genomes (Hunt *et al.* 2013b; Glastad *et al.* 2015). Knowledge of collinearity, however, is insufficient to demonstrate causality. Therefore, we sought to determine whether the correlation between dS and DNA methylation was the consequence of substitutions at CpG dinucleotides, where DNA methylation is predominantly targeted (Bird 1980; Bonasio *et al.* 2012).

**Figure 2:**
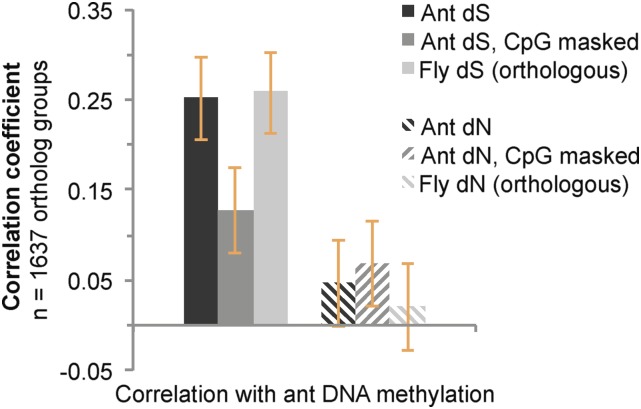
Correlations between *C. floridanus* DNA methylation and sequence substitution rates of ortholog groups in either ants or flies. Pearson’s correlation coefficients with 95% confidence intervals are shown.

We assessed correlations between DNA methylation in *C. floridanus* and dS among ants after masking positions with a CpG dinucleotide in any of the taxa included in multiple sequence alignments. Based on the hypothesis that DNA methylation causes an increase in both mutation rate and the rate of synonymous substitution, we predicted that we would not detect a significant correlation between DNA methylation and dS after masking CpG dinucleotides. Although the masking of CpG sites did indeed reduce the strength of correlation between dS and DNA methylation by 46%, a positive correlation between DNA methylation and dS persisted (Figure 2). One possible explanation for this finding is that neighboring methylated sites are subject to elevated mutation rates (Qu *et al.* 2012). However, the masking of CpG sites resulted in a reduction in correlations between dS and every factor we investigated in this study, by an average of 46% (Table S1). Thus, the reduced association between dS and DNA methylation when masking CpG sites may arise simply from an overall reduction of the signal-to-noise ratio of sequence alignments. As a result, we sought to gain further insight into the cause of the positive correlation between DNA methylation and dS by testing for an association between DNA methylation in *C. floridanus* and dS measured in *Drosophila* orthologs.

We predicted there would be no significant association between DNA methylation, as measured in *C. floridanus*, and orthologous dS measured among only *Drosophila* species, because DNA methylation does not exist at substantial levels in the genome of *D. melanogaster* or other flies (Urieli-Shoval *et al.* 1982; Zemach *et al.* 2010). Surprisingly, however, the strength of the positive correlation between *C. floridanus* DNA methylation and dS among ants did not differ significantly from the strength of the correlation between *C. floridanus* DNA methylation and dS calculated solely among *Drosophila* orthologs (Figure 2). This result provided evidence that DNA methylation is unlikely to be the dominant causal factor driving the elevated rate of synonymous substitutions observed for methylated genes in our study.

The possibility that other processes, besides DNA methylation, were responsible for the observed correlations with dS in insects is bolstered by an analysis of DNA methylation and substitution rate in introns of *Homo sapiens*, which revealed that DNA methylation level covaries with other factors that influence the overall substitution rate (Mugal and Ellegren 2011). However, in *H. sapiens*, DNA methylation was found to exhibit a strong influence on the CpG transition rate (Mugal and Ellegren 2011). We note that a more limited role in shaping variation in mutation rates may be expected for DNA methylation in insects and other invertebrates, as compared to vertebrate taxa, for at least two reasons. First, invertebrates exhibit substantially lower levels of DNA methylation than vertebrates (Zemach *et al.* 2010). Second, DNA methylation is often selectively localized to the 5′-region of genes in holometabolous insect taxa (Bonasio *et al.* 2012; Hunt *et al.* 2013a), while DNA methylation is globally targeted in the genomes of vertebrates (Suzuki and Bird 2008). What, then, is responsible for the elevated rates of synonymous substitutions observed for methylated genes in insects?

### DNA methylation is linked to chromatin states affecting coding sequence evolution

Recent studies have revealed that DNA methylation is integrated into domains of transcriptionally active chromatin in insect genomes (Nanty *et al.* 2011; Hunt *et al.* 2013b; Glastad *et al.* 2015). Thus, we chose to investigate whether combinatorial epigenetic states may explain the observed associations between coding sequence evolution and DNA methylation in *C. floridanus*. To this end, we performed a principal component analysis (PCA) of DNA methylation and seven histone modifications in *C. floridanus*, as well as another PCA of the same seven histone modifications in *D. melanogaster*. These analyses provided proxies for the assessment of distinct chromatin states among coding sequences by decomposing collinear epigenetic variables into independent components.

Three principal components (PCs) in each taxa explained greater than 10% of total variance in epigenetic marks, and the top three PCs together explained 76% and 82% of the total epigenetic variance in *C. floridanus* and *D. melanogaster*, respectively (Table 1). Among the top three PCs, DNA methylation loaded most heavily on PC1 in *C. floridanus* (Table 1). *C. floridanus* PC1 explained 39% of the total variance in epigenetic marks, and exhibited relatively large positive loadings of DNA methylation and three histone modifications associated with active transcription: H3K4me3, H3K36me3, and H3K27ac (“active” modifications; Kharchenko *et al.* 2011). Similarly, *D. melanogaster* PC1 explained 55% of the total variance in epigenetic marks, and also exhibited large positive loadings of these active histone modifications. In contrast, H3K9ac, which differed in its association with transcription in *C. floridanus* and *D. melanogaster* (Figure S3), negatively loaded on *C. floridanus* PC1, and positively loaded on *D. melanogaster* PC1. The histone modifications H3K9me3 and H3K27me3, which are associated with low transcriptional activity (“repressive” modifications; Kharchenko *et al.* 2011), both loaded negatively onto *D. melanogaster* PC1, while only H3K9me3 loaded negatively on *C. floridanus* PC1.

**Table 1:** Principal component (PC) analysis of epigenetic marks illustrates associations between chromatin state and coding sequence evolution

	*C. floridanus* (Ant)			*D. melanogaster* (Fly)		
	PC1 (39.4%)	PC2 (20.0%)	PC3 (16.6%)	PC1 (55.2%)	PC2 (14.8%)	PC3 (11.7%)
Eigenvectors						
DNA methylation	0.45	−0.33	−0.04	ND	ND	ND
H3K4me3	0.46	0.36	0.03	0.46	0.21	−0.12
H3K27ac	0.45	0.22	0.00	0.37	0.41	−0.16
H3K36me3	0.42	0.04	0.14	0.40	−0.33	0.40
H3K4me1	0.21	−0.39	0.45	0.37	−0.22	0.36
H3K27me3	0.02	0.18	0.77	−0.40	0.14	−0.25
H3K9ac	−0.04	0.72	−0.03	0.41	0.34	−0.41
H3K9me3	−0.40	0.07	0.43	−0.17	0.71	0.67
Correlation coefficients of gene expression metrics with PCs						
RNA Pol II	0.60****	0.07**	0.19****	0.78****	0.25****	−0.08***
Expression level	0.59****	0.01	−0.04	0.55****	0.02	0.00
Tissue specificity	ND	ND	ND	−0.60****	−0.05*	−0.01
Correlation coefficients of sequence substitution metrics with PCs						
dS	0.21****	−0.11****	−0.11****	0.20****	0.05*	0.10****
dN	−0.03	−0.15****	0.06**	−0.05*	−0.05*	0.07***
dN/dS	−0.11****	−0.12****	0.11****	−0.13****	−0.07***	0.04

PCs explaining less than 10% variation are not shown. *P < 0.05, **P < 10^–2^, ***P < 10^–3^, ****P < 10^–4^; ND, no data.

We found that PC1 exhibited striking positive correlations with both gene expression level and RNA Pol II occupancy in both taxa (Table 1). We also found that genes with high values of *C. floridanus* PC1 were significantly enriched for GO biological process terms related to cellular housekeeping functions, including ‘ribosome biogenesis’, ‘translation’, and ‘proton transport’ (Table S2). Accordingly, large PC1 values can be thought of as representing a transcriptionally active chromatin state in both taxa.

PC1 was also positively correlated with dS in both *C. floridanus* and *D. melanogaster* (Table 1). The positive correlation between PC1 and dS, coupled with the integration of DNA methylation into *C. floridanus* PC1, suggests that a transcriptionally active or “open” chromatin state may explain the bulk of the observed positive correlation between DNA methylation and dS in *C. floridanus* (Table 1).

**Figure 3:**
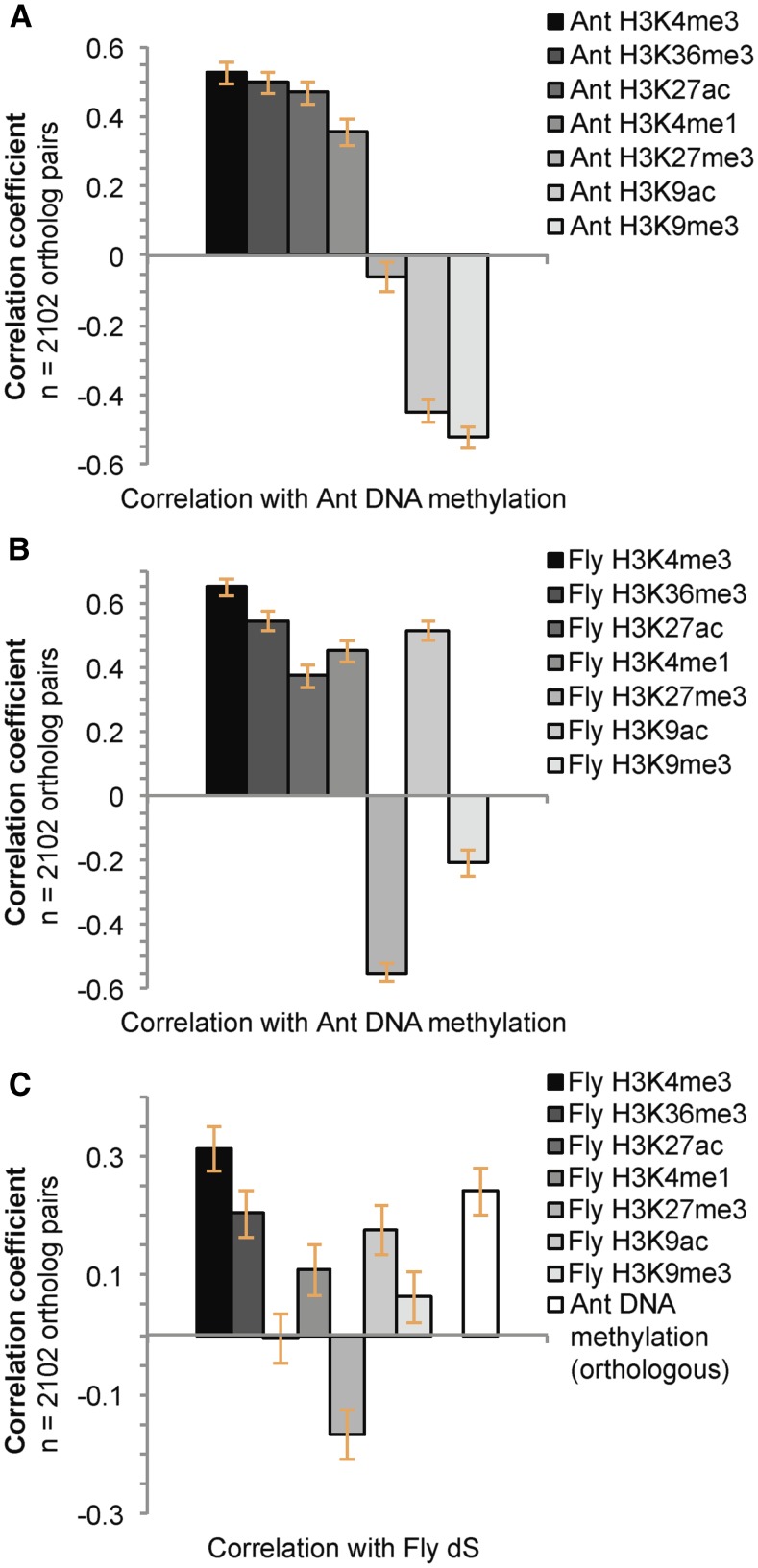
Correlations between DNA methylation and synonymous sequence substitution are mirrored by several histone modifications in insect genomes. (A) Correlations between histone modifications and DNA methylation in the ant *C. floridanus.* (B) Correlations between histone modifications in the fly *D. melanogaster* and orthologous DNA methylation in the ant *C. floridanus.* (C) Correlations between histone modifications in *D. melanogaster* and sequence substitution in flies mirror the relationship between *C. floridanus* DNA methylation and sequence substitution in flies. Pearson’s correlation coefficients with 95% confidence intervals are shown.

To further investigate the hypothesis that chromatin state was the critical factor affecting variation in rates of evolution in synonymous sites, we again leveraged the evolutionary loss of DNA methylation in *D. melanogaster*. We predicted that histone modifications in the genome of *D. melanogaster* that are (i) markers of transcriptionally active chromatin, and (ii) highly correlated with DNA methylation in the genome of *C. floridanus*, would be positively correlated with dS measures among *Drosophila* species (Figure 3, Table S4). Thus, we tested whether histone modifications that are correlated with DNA methylation levels in *C. floridanus* (Figure 3A; Glastad *et al.* 2015) were also correlated with dS in *D. melanogaster*, despite its absence of DNA methylation.

Remarkably, the two histone modifications that were most strongly correlated with DNA methylation in *C. floridanus*, H3K4me3 and H3K36me3, exhibited correlations with dS in *Drosophila* orthologs that did not differ significantly from the correlation between *Drosophila* dS and DNA methylation in *C. floridanus* orthologs (Figure 3C). We interpret this result as support for the hypothesis that loci residing in conserved, transcriptionally active chromatin domains (Engström *et al.* 2007; Hunt *et al.* 2013a) exhibit elevated rates of synonymous substitution in insect genomes, irrespective of the presence or absence of DNA methylation.

These findings raise the question of why genes residing in transcriptionally active chromatin would exhibit elevated synonymous substitution rates. One possible explanation is that genes residing in transcriptionally active chromatin exhibit elevated mutation rates resulting from the process of transcription itself. In support of this idea, a study of single-celled yeast and human germline cells recently revealed that mutation rates are positively correlated with gene expression level (Park *et al.* 2012). This suggests that eukaryotic transcription exerts a net mutagenic effect, in spite of transcription-coupled repair. Another possible explanation for elevated rates of synonymous substitution in regions of active chromatin is that selection acts more strongly on synonymous sites in regions of inaccessible chromatin *vs.* accessible chromatin, as suggested by an analysis of chromatin states and molecular evolution in *H. sapiens* (Prendergast *et al.* 2007). Finally, studies in yeast and mice have revealed that H3K4me3 is associated not only with active chromatin, but with recombination hotspots (Borde *et al.* 2009; Smagulova *et al.* 2011). Thus, it is feasible that GC-biased gene conversion, associated with meiotic recombination (Kent *et al.* 2012; Bolívar *et al.* 2015), also contributes to elevated synonymous substitution rates in genes targeted by H3K4me3 (but see Wallberg *et al.* 2015, which suggests DNA methylation may suppress recombination in the honey bee).

### Conclusions

We investigated how epigenetic marks, transcription, and gene structure relate to substitution rates in the genes of two highly diverged insect taxa. We found that DNA methylation was positively correlated with the rate of synonymous substitution. However, by comparing processes of molecular evolution in the presence and absence of DNA methylation, we revealed that this relationship was not explained primarily by the mutability of methylated cytosines in insects. Instead, the relationship between DNA methylation and synonymous substitution was apparently explained in large part by the targeting of DNA methylation to genes with signatures of transcriptionally active chromatin. We hypothesize that active chromatin may be prone to elevated rates of synonymous substitution by way of mutational pressures imposed by active transcription, or by differences in the structural requirements of distinct chromatin states. Overall, this research provides new insights into how epigenetic factors affect genome evolution in insects and other eukaryotic systems.

## Supplementary Material

Supporting Information
