# Supplementary material for: Effects of DNA Methylation and Chromatin State on Rates of Molecular Evolution in Insects
Source: G3 (Bethesda). 2015 Dec 2;6(2):357–63. doi: 10.1534/g3.115.023499 (PMC4751555; doi:10.1534/g3.115.023499)
Supplement: Supporting Information [file supp_6_2_357__index.html]

Effects of DNA Methylation and Chromatin State on Rates of Molecular Evolution in Insects — Supporting Information 

# Effects of DNA Methylation and Chromatin State on Rates of Molecular Evolution in Insects

## Supporting Information for Glastad *et al.*, 2016

**Files in this Data Supplement:**

- Figure S1 - Relationship between sequence substitution rate and gene characteristics according to Pearson's pairwise correlations in the ant *C. floridanus* and the fly *D. melanogaster*. (.pdf, 252 KB)
- Figure S2 - Relationship between sequence substitution rate and gene characteristics according to multiple linear regression models in the ant *C. floridanus* and the fly *D. melanogaster*. (.pdf, 245 KB)
- Figure S3 - Correlations between transcriptional activity and the histone modifications H3K4me3, H3K36me3, and H3K9ac. (.pdf, 118 KB)
- Table S1 - Pearson's correlations between *C. floridanus* gene characteristics and dS, as compared to correlations with dS after masking CpG sites. (.pdf, 28 KB)
- Table S2 - Gene ontology annotation enrichment of genes with the highest 300 values for each *Camponotus floridanus* principal component (from the analysis summarized in Table 1) relative to all other genes in our principal component analysis. (.pdf, 33 KB)
- Table S3 - Similarities and differences in associations with coding sequence evolution in an ant (*C. floridanus*) and a fly (*D. melanogaster*) in linear models generated from traits with data in both taxa, limited to common orthologs (n = 2102). (.pdf, 31 KB)
- Table S4 - Pearson's correlations between *C. floridanus* and *D. melanogaster* orthologs (n = 2102 ortholog pairs) for traits examined in this study. (.pdf, 27 KB)
- File S1 - File contains Supplementary Text and Supplementary References. (.pdf, 57 KB)
- File S2 - Transformed data. (.xlsx, 4,165 KB)
