# Supplementary material for: Effects of DNA Methylation and Chromatin State on Rates of Molecular Evolution in Insects
Source: G3 (Bethesda). 2015 Dec 2;6(2):357–63. doi: 10.1534/g3.115.023499 (PMC4751555; doi:10.1534/g3.115.023499)
Supplement: Supporting Information [file supp_g3.115.023499_FigureS1.pdf]

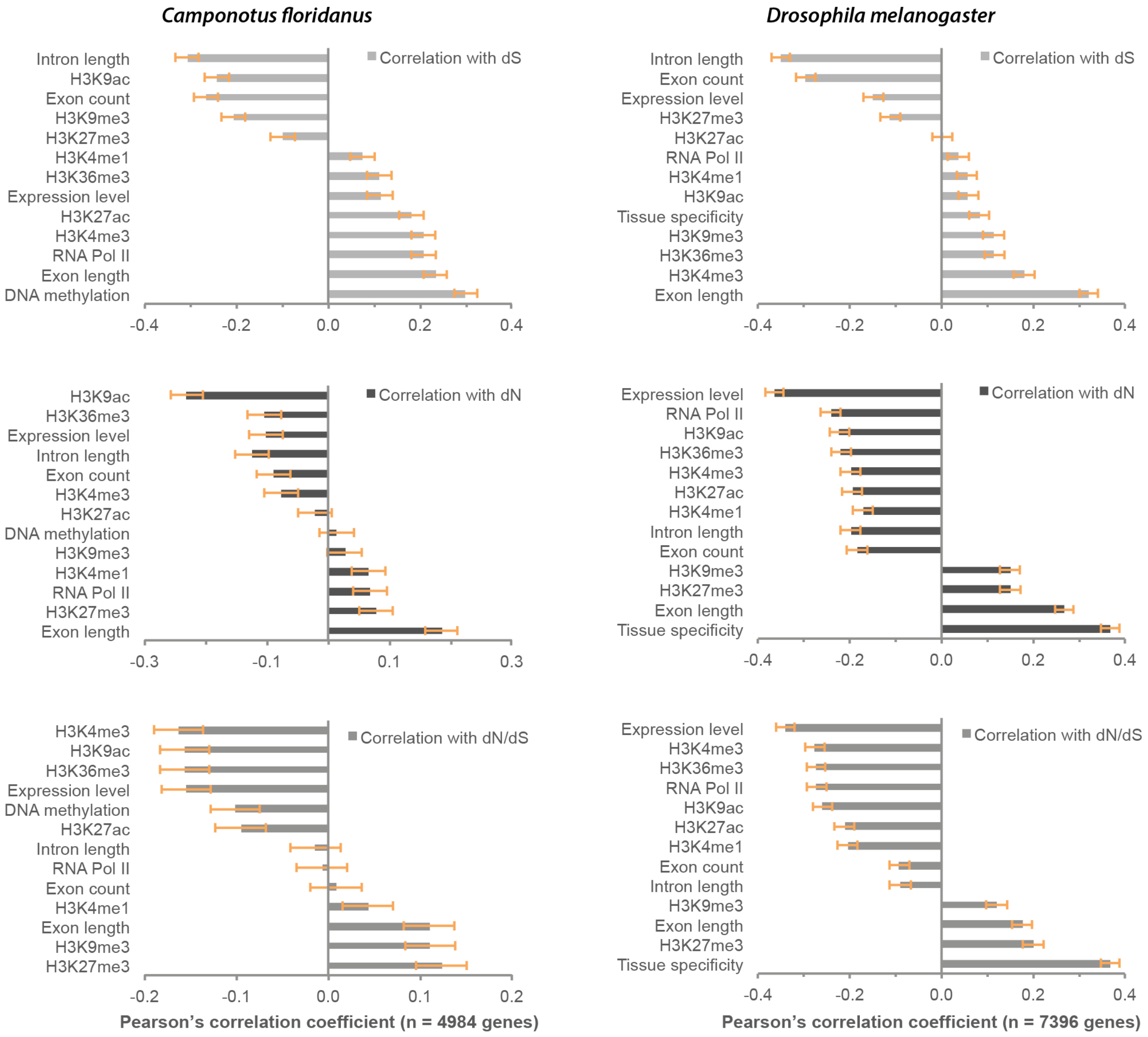

Figure S1. Relationship between sequence substitution rate and gene characteristics according to Pearson's pairwise correlations in the ant *C. floridanus* and the fly *D. melanogaster*. Correlation coefficients are plotted with 95% confidence intervals.
