# Supplementary material for: Effects of DNA Methylation and Chromatin State on Rates of Molecular Evolution in Insects
Source: G3 (Bethesda). 2015 Dec 2;6(2):357–63. doi: 10.1534/g3.115.023499 (PMC4751555; doi:10.1534/g3.115.023499)
Supplement: Supporting Information [file supp_g3.115.023499_FigureS3.pdf]

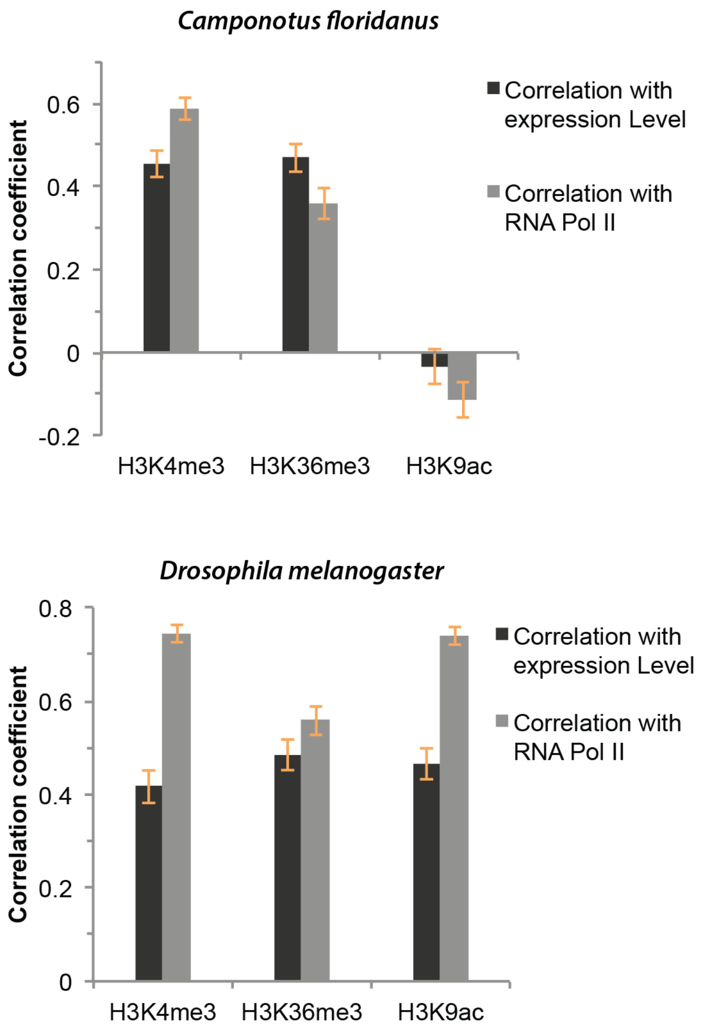

Figure S3. Correlations between transcriptional activity and the histone modifications H3K4me3, H3K36me3, and H3K9ac. Pearson's correlations with 95% confidence intervals are shown for data from *C. floridanus* and *D. melanogaster* (n = 2102 common ortholog groups).
