# Supplementary material for: Effects of DNA Methylation and Chromatin State on Rates of Molecular Evolution in Insects
Source: G3 (Bethesda). 2015 Dec 2;6(2):357–63. doi: 10.1534/g3.115.023499 (PMC4751555; doi:10.1534/g3.115.023499)
Supplement: Supporting Information [file supp_g3.115.023499_TableS1.pdf]

Table S1. Pearson's correlations between *C. floridanus* gene characteristics and dS, as compared to correlations with dS after masking CpG sites

X	***X*** correlation with ant dS	***X*** correlation with ant dS, CpGs masked	Percent decrease in correlation after CpG masking	P-value, dS correlation	P-value, dS, CpGs masked correlation
DNA methylation	0.28	0.15	46%	< 0.0001	< 0.0001
Exon length (mean)	0.28	0.20	27%	< 0.0001	< 0.0001
H3K4me3	0.19	0.14	29%	< 0.0001	< 0.0001
RNA Pol II	0.17	0.13	25%	< 0.0001	< 0.0001
H3K27ac	0.15	0.10	34%	< 0.0001	< 0.0001
Expression level	0.08	0.05	31%	< 0.0001	0.0008
H3K36me3	0.05	0.00	93%	0.002	0.8225
H3K4me1	0.05	0.01	81%	0.0047	0.5926
H3K27me3	-0.09	-0.03	68%	< 0.0001	0.066
H3K9me3	-0.17	-0.07	57%	< 0.0001	< 0.0001
H3K9ac	-0.21	-0.11	48%	< 0.0001	< 0.0001
Intron length (mean)	-0.30	-0.18	41%	< 0.0001	< 0.0001
Exon count	-0.32	-0.26	18%	< 0.0001	< 0.0001
