# Supplementary material for: Effects of DNA Methylation and Chromatin State on Rates of Molecular Evolution in Insects
Source: G3 (Bethesda). 2015 Dec 2;6(2):357–63. doi: 10.1534/g3.115.023499 (PMC4751555; doi:10.1534/g3.115.023499)
Supplement: Supporting Information [file supp_g3.115.023499_TableS2.pdf]

Table S2. Gene ontology annotation enrichment of genes with the highest 300 values for each *Camponotus floridanus* principal component (from the analysis summarized in Table 1) relative to all other genes in our principal component analysis.

PC	Term	Category[a]	Accession	No. genes (of 300)	Fold-enriched	FDR *P*-value
PC1	structural constituent of ribosome	F	GO:0003735	17	6.0	7.74E-04
	ribosome biogenesis	P	GO:0042254	19	4.3	2.43E-03
	proteasome complex	C	GO:0000502	10	8.1	2.82E-03
	translation	P	GO:0006412	27	2.8	5.28E-03
	actin polymerization or depolymerization	P	GO:0008154	7	12.4	6.13E-03
	proton transport	P	GO:0015992	10	6.7	6.13E-03
	oxidative phosphorylation	P	GO:0006119	9	6.6	1.16E-02
	cytosolic ribosome	C	GO:0022626	3	NA[b]	3.72E-02
	respiratory chain complex	C	GO:0098803	6	9.5	3.72E-02
	hydrogen ion transmembrane transporter activity	F	GO:0015078	8	5.8	4.79E-02
PC2	none significant at FDR $P < 0.05$					
PC3	sequence-specific DNA binding	F	GO:0043565	19	3.8	1.72E-02
	aromatic compound biosynthetic process	P	GO:0019438	48	2.2	1.72E-02
	cellular nitrogen compound biosynthetic process	P	GO:0044271	48	2.2	1.72E-02
	heterocycle biosynthetic process	P	GO:0018130	48	2.1	1.72E-02
	organic cyclic compound biosynthetic process	P	GO:1901362	49	2.1	1.72E-02
	regulation of transcription, DNA-templated	P	GO:0006355	26	2.8	1.72E-02
	sequence-specific DNA binding transcription factor activity	F	GO:0003700	21	3.0	2.66E-02
	regulation of transcription, DNA-templated	P	GO:0006355	32	2.3	2.99E-02

[a] P, biological process; F, molecular function; C, cellular component

[b] No genes with this term were present in the reference set, preventing calculation of fold enrichment.
