# Supplementary material for: Effects of DNA Methylation and Chromatin State on Rates of Molecular Evolution in Insects
Source: G3 (Bethesda). 2015 Dec 2;6(2):357–63. doi: 10.1534/g3.115.023499 (PMC4751555; doi:10.1534/g3.115.023499)
Supplement: Supporting Information [file supp_g3.115.023499_TableS3.pdf]

Table S3. Similarities and differences in associations with coding sequence evolution in an ant (*C. floridanus*) and a fly (*D. melanogaster*) in linear models generated from traits with data in both taxa, limited to common orthologs (n = 2102)

	C. floridanus **model coefficient**	***D. melanogaster model coefficient***	**Consistent significant relationship with *X* between species?**
X	**dS (R^2 = 0.26)**	**dS (R^2 = 0.34)**	
H3K9ac	-0.38****	-0.08	No
Exon count	-0.20****	0.07*	No
H3K4me1	-0.06*	-0.15****	Yes, negative
H3K27me3	-0.04	-0.03	No
H3K36me3	-0.03	0.17****	No
Intron length	-0.02	-0.13****	No
Expression level	0.01	-0.27****	No
H3K9me3	0.03	0.10****	No
H3K27ac	0.04	-0.01	No
RNA Pol II	0.09**	-0.09**	No
H3K4me3	0.20****	0.52****	Yes, positive
Exon length	0.26****	0.29****	Yes, positive
X	**dN (R^2 = 0.16)**	**dN (R^2 = 0.15)**	
H3K9ac	-0.26****	0.05	No
H3K36me3	-0.15****	-0.05	No
Expression level	-0.07**	-0.25****	Yes, negative
H3K4me3	-0.04	0.09	No
H3K9me3	-0.03	0.03	No
Intron length	0.01	-0.06*	No
H3K4me1	0.04	-0.01	No
Exon count	0.06	0.21****	No
H3K27ac	0.12**	-0.01	No
RNA Pol II	0.14****	-0.04	No
H3K27me3	0.17****	-0.15****	No
Exon length	0.36****	0.35****	Yes, positive
X	**dN/dS (R^2 = 0.12)**	**dN/dS (R^2 = 0.09)**	
H3K36me3	-0.15****	-0.11**	Yes, negative
H3K9ac	-0.14****	0.08	No
H3K4me3	-0.12**	-0.10	Yes, negative
Expression level	-0.08**	-0.17****	Yes, negative
H3K9me3	-0.04	0.00	No
Intron length	0.02	-0.02	No
H3K4me1	0.07*	0.04	No
RNA Pol II	0.11**	0.00	No
H3K27ac	0.11**	0.00	No
Exon count	0.13****	0.20****	Yes, positive
H3K27me3	0.20****	-0.14****	No
Exon length	0.30****	0.27****	Yes, positive

*P < 0.05, **P < 10-2, ***P < 10-3, ****P < 10-4
