# Supplementary material for: Effects of DNA Methylation and Chromatin State on Rates of Molecular Evolution in Insects
Source: G3 (Bethesda). 2015 Dec 2;6(2):357–63. doi: 10.1534/g3.115.023499 (PMC4751555; doi:10.1534/g3.115.023499)
Supplement: Supporting Information [file supp_g3.115.023499_TableS4.pdf]

Table S4. Pearson's correlations between *C. floridanus* and *D. melanogaster* orthologs (n = 2102 ortholog pairs) for traits examined in this study

X	Correlation between species
dN	0.65****
dN/dS	0.61****
Expression level	0.56****
H3K4me3	0.55****
Exon count	0.53****
H3K27ac	0.52****
H3K36me3	0.51****
RNA Pol II	0.46****
Exon length (mean)	0.43****
dS	0.36****
Intron length (mean)	0.28****
H3K9me3	0.22****
H3K4me1	0.19****
H3K27me3	0.12****
H3K9ac	-0.08***

***P < 10-3, ****P < 10-4
