# Supplementary material for: Effects of DNA Methylation and Chromatin State on Rates of Molecular Evolution in Insects
Source: G3 (Bethesda). 2015 Dec 2;6(2):357–63. doi: 10.1534/g3.115.023499 (PMC4751555; doi:10.1534/g3.115.023499)
Supplement: Supporting Information [file supp_g3.115.023499_FileS1.pdf]

File S1. SUPPLEMENTARY MATERIAL

Effects of DNA methylation and chromatin state on rates of molecular evolution in insects

Karl M. Glastad, Michael A. D. Goodisman, Soojin V. Yi, Brendan G. Hunt

Supplementary text: Common factors associated with coding sequence evolution in two insect orders

Our investigation into genetic and epigenetic factors associated with rates of coding sequence evolution provided insight into the factors that shape evolutionary rate variation in two insect orders, the Diptera and the Hymenoptera, that diverged approximately 350 Ma (WIEGMANN *et al.* 2009). In order to directly assess common factors associated with variation in the evolutionary rates of coding sequences, we generated multiple linear regression models using only those characteristics of orthologs for which data were present in both *C. floridanus* and *D. melanogaster* (Table S3). We found that average exon length was positively associated with both nonsynonymous substitution rate (dN) and synonymous substitution rate (dS) in *C. floridanus* and *D. melanogaster* (Table S3). This previously discovered relationship may be explained by the associations of mean exon size with gene expression breadth (DURET AND MOUCHIROUD 2000; EISENBERG AND LEVANON 2003) and nucleosome positioning (SCHWARTZ *et al.* 2009; PRENDERGAST AND SEMPLE 2011; cf. LAWRIE *et al.* 2013). Similarly, average exon length and the number of exons in a gene were each positively associated with dN/dS when considered in a multiple regression framework (Table S3), consistent with selection for compactness operating on highly conserved genes (EISENBERG AND LEVANON 2003).

We found that gene expression level was negatively associated with dN and dN/dS in both *C. floridanus* and *D. melanogaster* (Table S3). This relationship has been widely observed and may be attributable to selection against protein mistranslation (DURET AND MOUCHIROUD 1999; DRUMMOND *et al.* 2006; DRUMMOND AND WILKE 2008). Similarly, two histone modifications associated with active transcription, H3K4me3 and H3K36me3 (KHARCHENKO *et al.* 2011; ZHOU *et al.* 2011), were negatively associated with dN/dS. Moreover, H3K4me3 was positively associated with dS in both taxa (Table S3), suggesting this epigenetic mark may be linked to variation in mutation rate or structural constraints on chromatin (PRENDERGAST *et al.* 2007; PARK *et al.* 2012).

Supplementary References

Drummond, D. A., A. Raval and C. O. Wilke, 2006 A single determinant dominates the rate of yeast protein evolution. Molecular Biology and Evolution 23**:** 327-337.

Drummond, D. A., and C. O. Wilke, 2008 Mistranslation-induced protein misfolding as a dominant constraint on coding-sequence evolution. Cell 134**:** 341-352.

Duret, L., and D. Mouchiroud, 1999 Expression pattern and, surprisingly, gene length shape codon usage in Caenorhabditis, Drosophila, Arabidopsis. Proceedings of the National Academy of Sciences of the United States of America 96**:** 4482-4487.

Duret, L., and D. Mouchiroud, 2000 Determinants of substitution rates in mammalian genes: expression pattern affects selection intensity but not mutation rate. Molecular Biology and Evolution 17**:** 68-74.

Eisenberg, E., and E. Y. Levanon, 2003 Human housekeeping genes are compact. Trends in Genetics 19**:** 362-365.

Kharchenko, P. V., A. A. Alekseyenko, Y. B. Schwartz, A. Minoda, N. C. Riddle *et al.*, 2011 Comprehensive analysis of the chromatin landscape in *Drosophila melanogaster*. Nature 471**:** 480-485.
Lawrie, D. S., P. W. Messer, R. Hershberg and D. A. Petrov, 2013 Strong purifying selection at synonymous sites in *D. melanogaster*. PLoS Genet 9**:** e1003527.
Park, C., W. Qian and J. Zhang, 2012 Genomic evidence for elevated mutation rates in highly expressed genes. EMBO reports 13**:** 1123-1129.
Prendergast, J., H. Campbell, N. Gilbert, M. Dunlop, W. Bickmore *et al.*, 2007 Chromatin structure and evolution in the human genome. BMC Evolutionary Biology 7**:** 72.
Prendergast, J. G. D., and C. A. M. Semple, 2011 Widespread signatures of recent selection linked to nucleosome positioning in the human lineage. Genome Research 21**:** 1777-1787.
Schwartz, S., E. Meshorer and G. Ast, 2009 Chromatin organization marks exon-intron structure. Nature Structural & Molecular Biology 16**:** 990-995.
Wiegmann, B., M. Trautwein, J.-W. Kim, B. Cassel, M. Bertone *et al.*, 2009 Single-copy nuclear genes resolve the phylogeny of the holometabolous insects. BMC Biology 7**:** 34.
Zhou, V. W., A. Goren and B. E. Bernstein, 2011 Charting histone modifications and the functional organization of mammalian genomes. Nature Reviews Genetics 12**:** 7-18.
